# 

**Published:** 1889-07

**Authors:** 


### CONTENTS:

Article I.—Dental Decay. By W. D. Miller. . . . 97

" II.—Dental Irregularities of the Native Races. By E. L. Townsend, D. D. S. . . . . 100

" III.—A Surgeon's Notes on Certain Diseases and Malformations of the Teeth. By Jonathan Hutchinson, F. R. S., LL. D. . . . . 104

" IV.—Restoring Discolored Teeth. By W. A. Barrows, D. D. S. . . . . 112

" V.—Progress of Dentistry During Year Ending May 1st, 1889. By Rodrigues Ottolengui. 117

" VI.—A Study of Arsenious Acid. By L. Ashley Faught, D. D. S. . . . . 132

### EDITORIAL.

University of Maryland Dental Department. . . . . 138

Saccharine. . . . . . . . . . . . . . 138

A Victim of Cocaine. . . . . . . . . . . . 139

### OBITUARY.

F. H. Rehwinkel, M. D., D. D. S. . . . . . . . . 140

### BIBLIOGRAPHICAL.

Dental Medicine. . . . . . . . . . . . . 142

The Principles and Practice of Dentistry. . . . . . 143

### MONTHLY SUMMARY.

Filling Teeth With Vulcanite. . . . . . . . . 144

